# Supplementary material for: Differential controls of MAIT cell effector polarization by mTORC1/mTORC2 via integrating cytokine and costimulatory signals
Source: Nat Commun. 2021 Apr 1;12:2029. doi: 10.1038/s41467-021-22162-8 (PMC8016978; doi:10.1038/s41467-021-22162-8)
Supplement: Supplementary file 1 — Supplementary Information [file 41467_2021_22162_MOESM1_ESM.pdf]

Supplemental Figures

Differential controls of MAIT cell effector polarization by mTORC1/mTORC2 via integrating cytokine and costimulatory signals

Huishan Tao[#], Yun Pan[#], Shuai Chu[#], Lei Li[#], Jinhai Xie, Peng Wang, Shimeng Zhang,

Srija Reddy, John W. Sleasman, and Xiao-Ping Zhong*

[#]Cofirst authors

* Corresponding author

Supplemental Figure 1

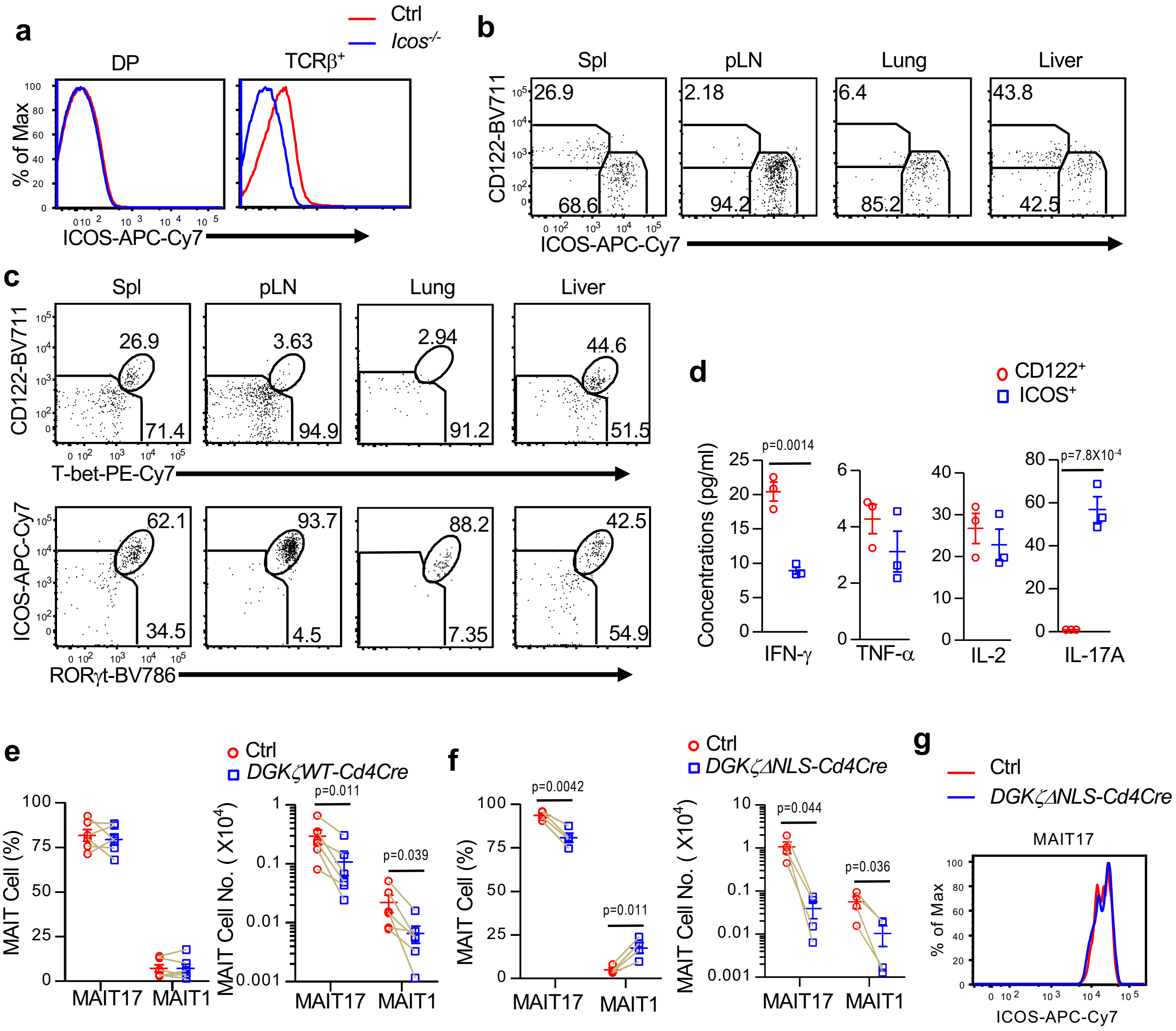

Supplemental Figure 1. Defining MAIT1 and MAIT17 effector cells by ICOS and CD122 expression and their regulation by DGKζ. a. ICOS expression in TCRβ⁻CD4⁺CD8⁺ and TCRβ⁺ thymocytes from WT and *Icos*⁻/⁻ mice. **b.** ICOS vs CD122 FACS plots in CD24⁻CD44⁺ MAIT cells from indicated organs in WT mice. **c.** CD122 vs T-bet and ICOS vs RORγt FACS plots in CD24⁻CD44⁺ MAIT cells from indicated organs in WT mice. **d.** Cytokines in cultural supernatants of sorted MAIT1 and MAIT17 cells after PMA and ionomycin stimulation for 4 hours detected by a mulplex-ELISA (n = 3). **e.** Thymic MAIT1 and MAIT17 percentages and numbers in *Dgkz*^{WT}*-Cd4Cre* and WT control mice. **f.** Thymic MAIT1 and MAIT17 percentages and numbers in *Dgkz*$^{\Delta NLS}$*-Cd4Cre* and WT control mice. **g.** ICOS expression in thymic MAIT17 cells from WT control and *Dgkz*$^{\Delta NLS}$*-Cd4Cre* mice. Data shown are representative of or are pooled from at least three experiments. Bars represent mean ± SEM. Statistical significance is determined by two-tailed unpaired Student t-test for d and pairwise Student t-test for e and f. P values of less than 0.05 are shown. Source data for all graphs are provided as a Source Data file.

Supplemental Figure 2

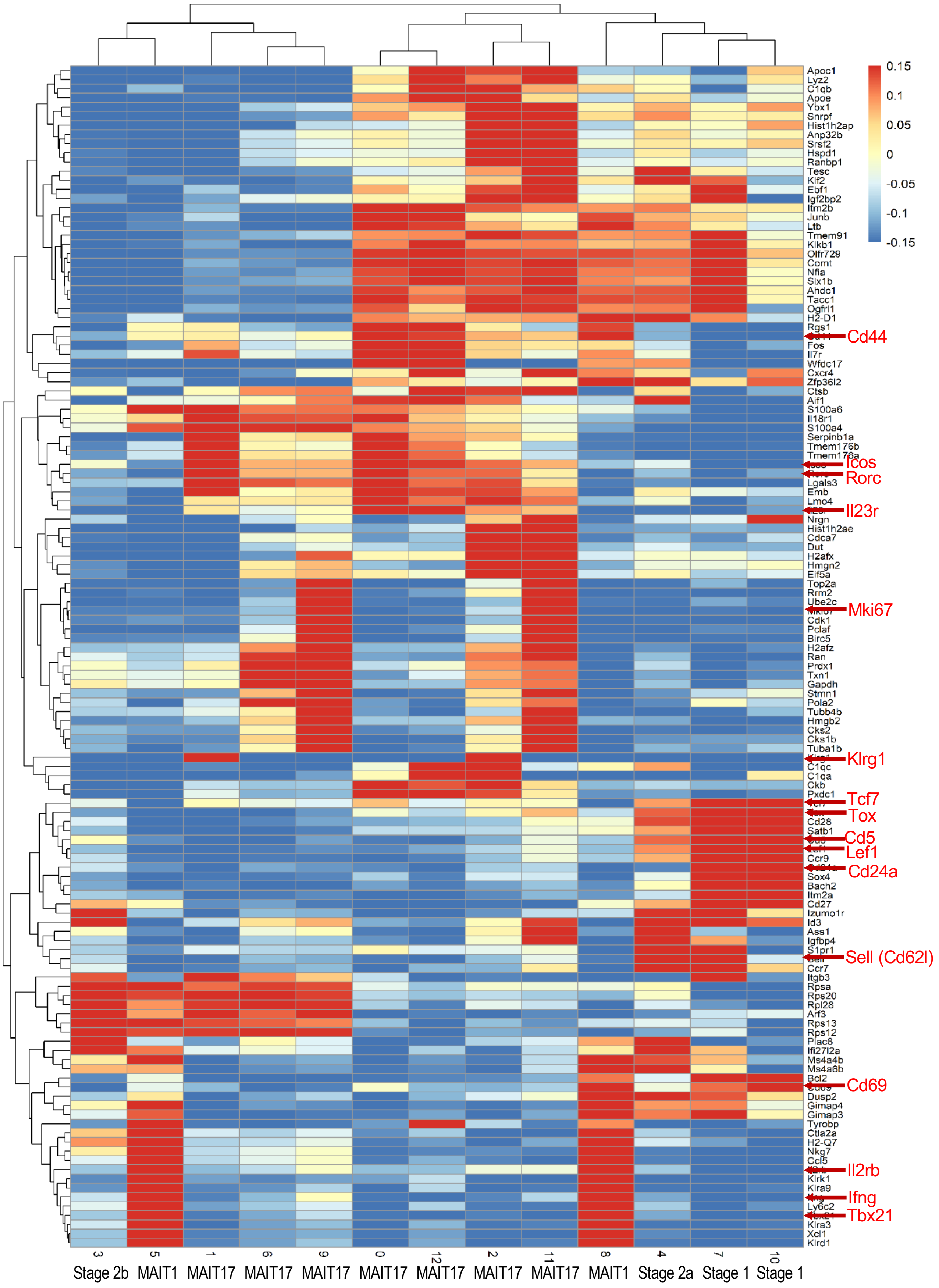

Supplemental Figure 2. Differential expression of characteristic genes in different clusters of WT thymic MAIT cells based on scRNAseq analysis. Heat-map represents expression of top 10 upregulated genes in each MAIT cell clusters shown in Figure 1g and a selected list of genes (*Sell*, *Cd69*, *S1pr1*, *Cd5*, *Ccr7*, *Itm2a*, *Cd24a*, *Cd44*, *Klf2*, *Tcf7*, *Bach2*, *Satb1*, *Lef1*, *Ebf1*, *Tbx21*, *Rorc*, *Ifng*, *Il23r*, *Icos*, *Il2rb*, *Mki67*, *Cxcr4*, *Klrg1*).

Supplemental Figure 3

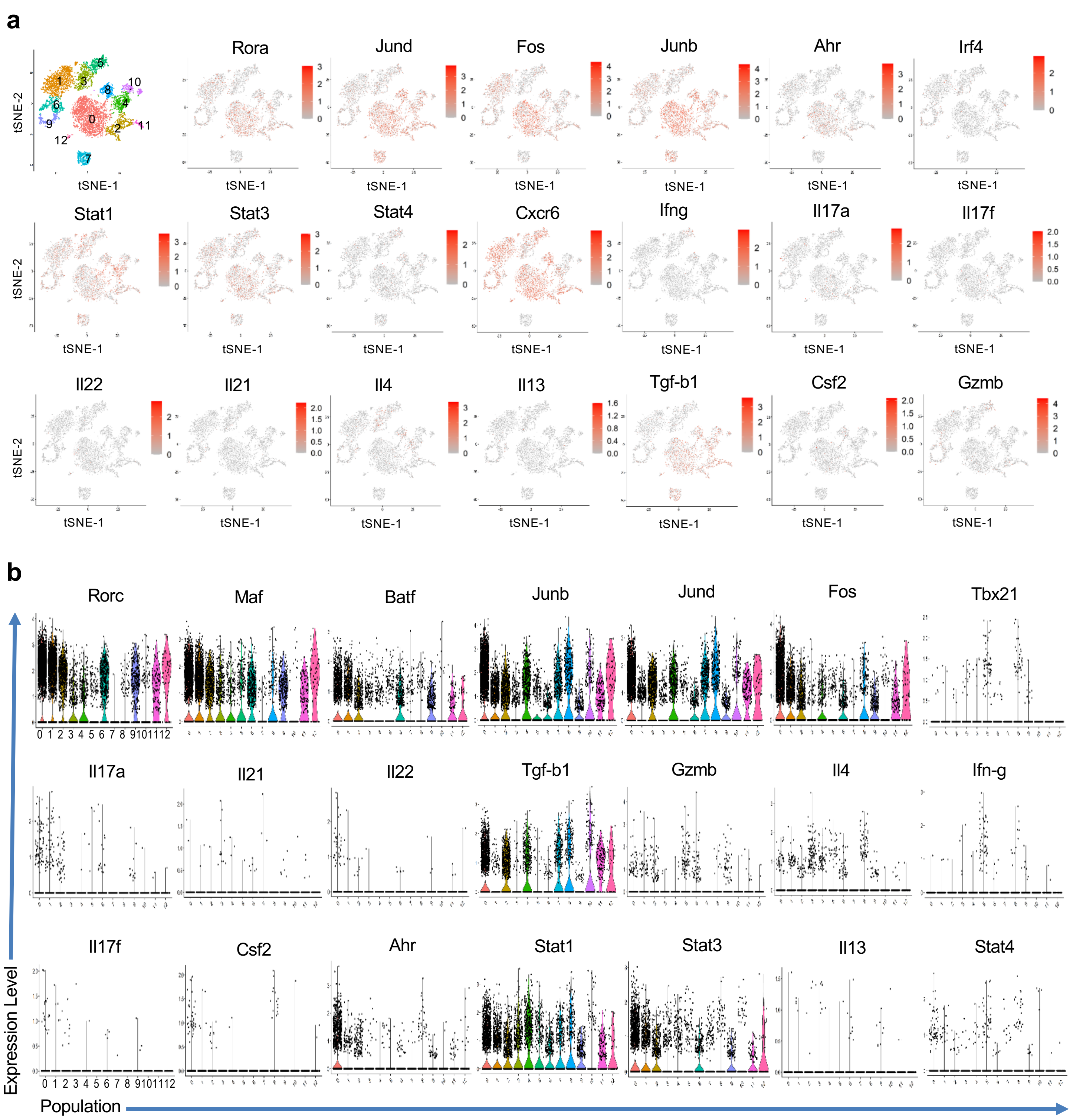

Supplemental Figure 3. Expression of effector lineage related genes in WT thymic MAIT cell clusters from scRNAseq analysis. a. tSNE plots show expression of indicated genes in different clusters. **b.** Violin plots show expression of indicated genes in MAIT cell clusters.

Supplemental Figure 4

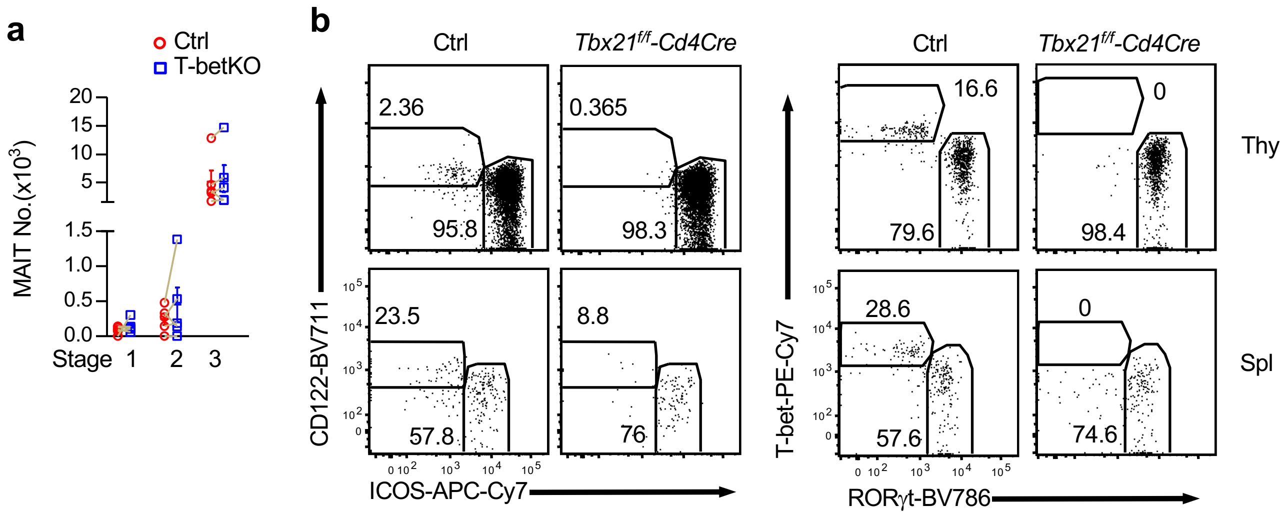

Supplemental Figure 4. Analysis of T-bet deficient mice. Eight – ten weeks old WT and *Tbx21^{f/f}-Cd4Cre* mice were analyzed. **a.** Stage 1 – 3 MAIT cell numbers in the thymus. **b.** Representative FACS plots showing CD122 vs ICOS and RORγt vs T-bet expression in thymic (enriched) and splenic MAIT cells. Data shown were representative of or pooled from five experiments. Source data for the graph is provided as a Source Data file.

Supplemental Figure 5

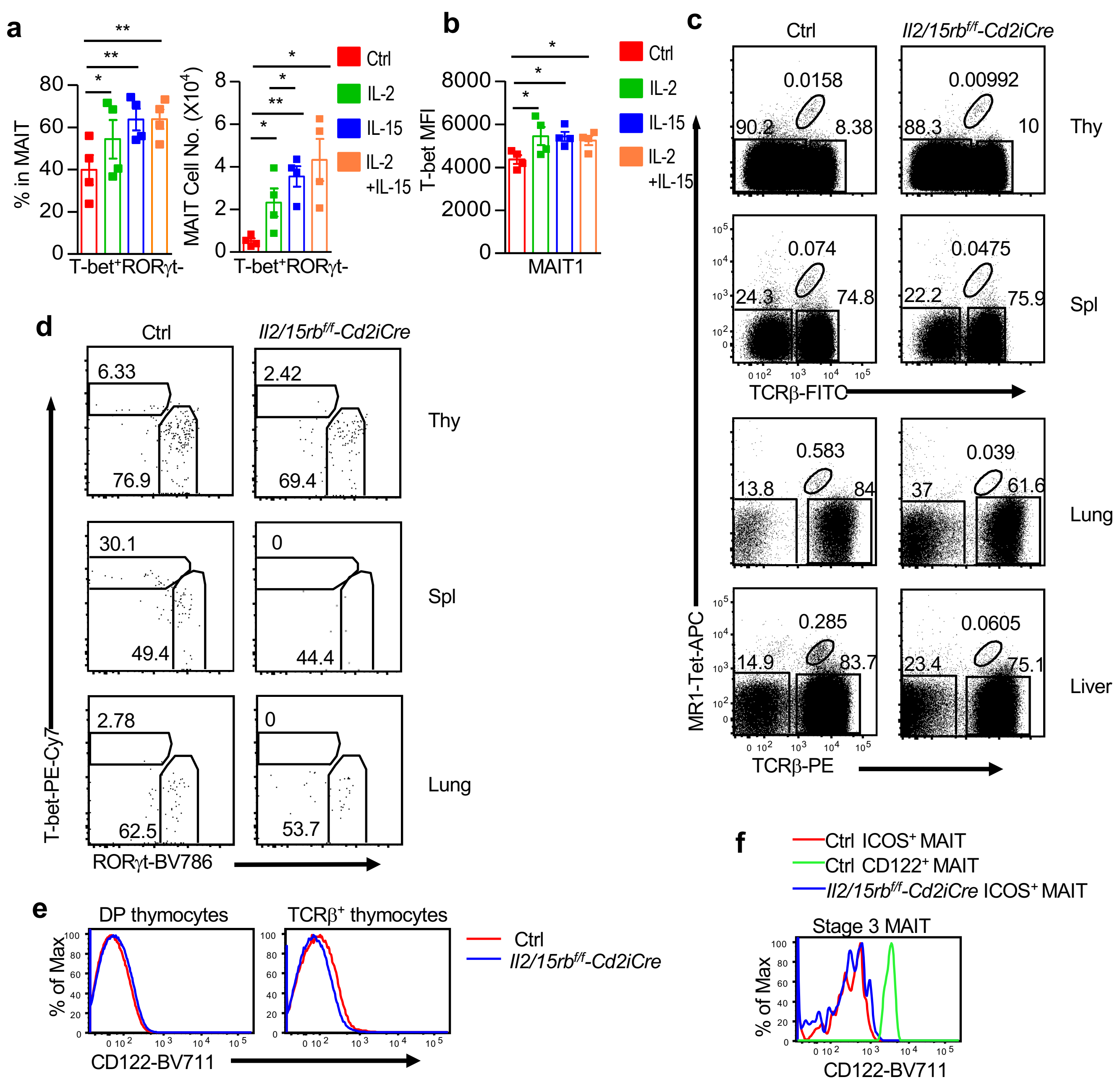

Supplemental Figure 5. Role of IL-2/IL-15R signal in MAIT cells.
a, b. Splenic MAIT1 cells in WT mice injected with either IL-2/anti-IL-2 complex, IL-15/IL-15RFc, or both. **a.** T-bet^{+}RORγt^{-} MAIT1 cell percentages and numbers. **b.** T-bet MFI in MAIT1 cells. Data show were pooled from four experiments. *, $p<0.05$; **, $p<0.01$ are determined by two-tailed pairwise Student t-test. Source data for all graphs are provided as a Source Data file.
c – f. Analysis of MAIT cells in *Il2/15rb$^{f/f}$-Cd2iCre* mice. **c.** Representative FACS plots of MAIT cell staining in *Il2/15rb$^{f/f}$-Cd2iCre* and WT control mice. **d.** Representative FACS plots showing T-bet and RORγt staining in stage 3 MAIT cells in the indicated organs. **e.** Overlaid histogram shows CD122 expression in TCRβ^{-} DP and TCRβ^{+} SP thymocytes. **f.** Overlaid histogram shows CD122 expression in CD122^{-}ICOS^{+} and CD122^{+}ICOS^{low} stage 3 MAIT cells from WT control mice, and ICOS^{+} MAIT cells from *Il2/15rb$^{f/f}$-Cd2iCre* mice. Data are representative of at least five experiments.

Supplemental Figure 6

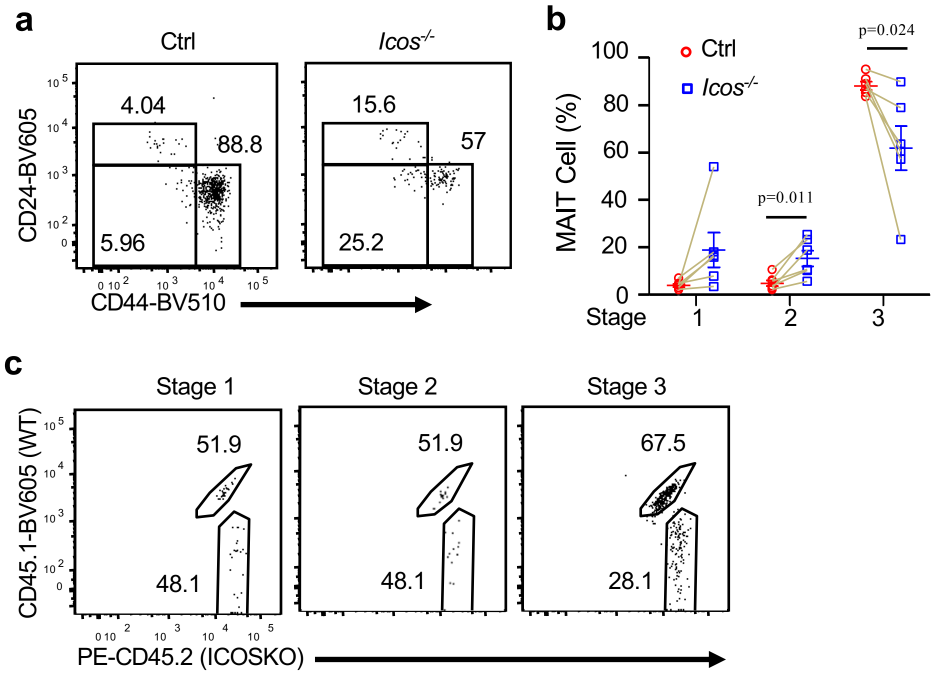

Supplemental Figure 6. Effects of ICOS deficiency on MAIT cells. a. Representative FACS plots show CD24 and CD44 staining of thymic MAIT cells from WT and ICOSKO mice. **b.** Scatter plots of percentages of stages 1 – 3 thymic MAIT cells from WT and ICOSKO mice. **c.** Representative FACS plots show distribution of $CD45.1^{+}CD45.2^{+}$ WT and $CD45.1^{-}CD45.2^{+}$ ICOSKO cells within stage 1, 2, and 3 MAIT cells in mixed BM chimeric mice. Data shown are representative of or pooled from six experiments for a and b and four experiments for c. Statistical significance is determined by two-tail pairwise Student t-test. P values of less than 0.05 are shown. Source data for the graph is provided as a Source Data file.

Supplemental Figure 7

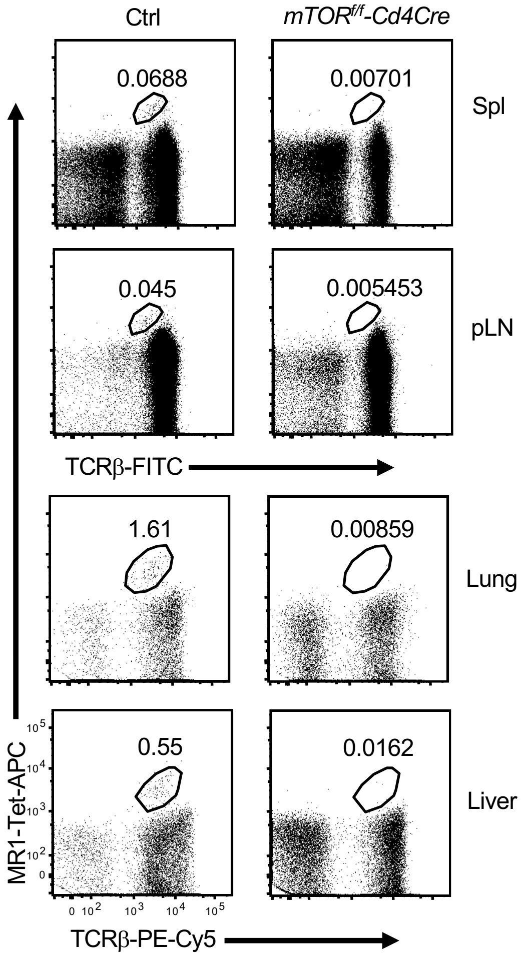

Supplemental Figure 7. Analysis of MAIT cells in *mTOR^f/f^-Cd4Cre* mice. Eight – ten weeks old *mTOR^f/f^-Cd4Cre* and control mice were analyzed. Representative FACS plots of MAIT cell staining in the indicated organs. Data shown are representative of at least five experiments.

Supplemental Figure 8

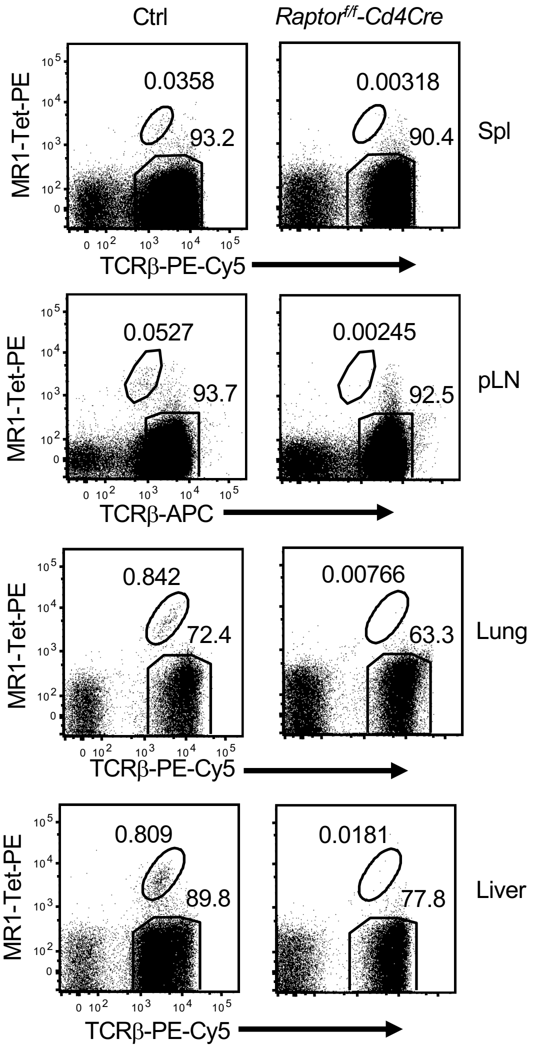

Supplemental Figure 8. Analysis of MAIT cells in *Raptor$^{f/f}$-Cd4Cre* mice. Eight – ten weeks old *Raptor$^{f/f}$-Cd4Cre* and control mice were analyzed. Representative FACS plots of MAIT cell staining in the indicated organs. Data shown are representative of seven experiments.

Supplemental Figure 9

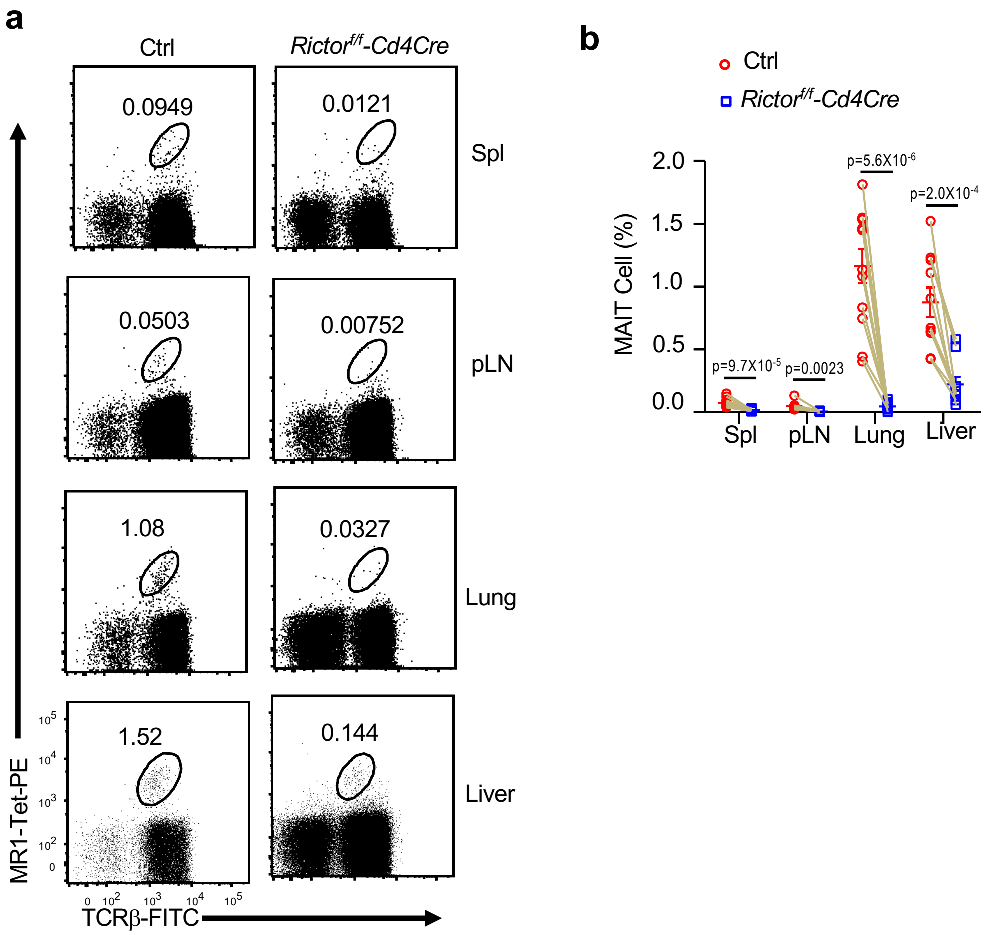

Supplemental Figure 9. Analysis of MAIT cells in *Rictor^f/f^-Cd4Cre* mice. Eight – ten weeks old *Rictor^f/f^-Cd4Cre* and control mice were analyzed. **a.** Representative FACS plots of MAIT cell staining in the indicated organs. **b.** MAIT cell percentages in peripheral organs. Data shown are representative of or pooled from at least nine experiments. Statistical significance is determined by two-tailed pairwise Student t-test. P values of less than 0.05 are shown. Source data for all graphs are provided as a Source Data file.

Supplemental Figure 10

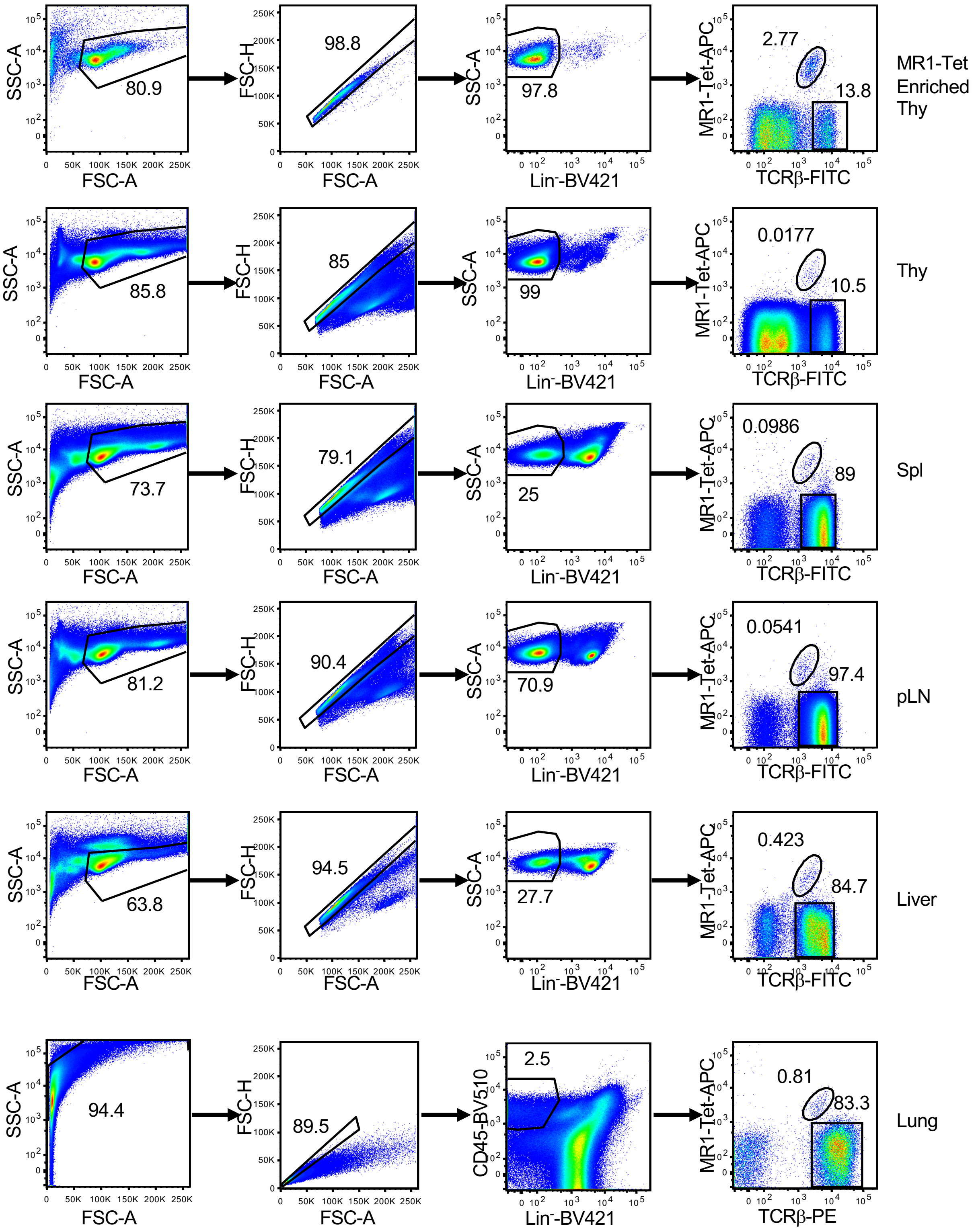

Supplemental Figure 10. Gating strategies for MAIT cells.
