# Supplementary material for: Differential controls of MAIT cell effector polarization by mTORC1/mTORC2 via integrating cytokine and costimulatory signals
Source: Nat Commun. 2021 Apr 1;12:2029. doi: 10.1038/s41467-021-22162-8 (PMC8016978; doi:10.1038/s41467-021-22162-8)
Supplement: Supplementary file 3 — Reporting Summary [file 41467_2021_22162_MOESM3_ESM.pdf]

nature research

Corresponding author(s): Xiao-Ping Zhong

Last updated by author(s): 2021-2-23

Reporting Summary

Nature Research wishes to improve the reproducibility of the work that we publish. This form provides structure for consistency and transparency in reporting. For further information on Nature Research policies, see our Editorial Policies and the Editorial Policy Checklist.

Statistics

For all statistical analyses, confirm that the following items are present in the figure legend, table legend, main text, or Methods section.

n/a	Confirmed	
☐	☒	The exact sample size (*n*) for each experimental group/condition, given as a discrete number and unit of measurement
☐	☒	A statement on whether measurements were taken from distinct samples or whether the same sample was measured repeatedly
☐	☒	The statistical test(s) used AND whether they are one- or two-sided<br>*Only common tests should be described solely by name; describe more complex techniques in the Methods section.*
☐	☒	A description of all covariates tested
☒	☐	A description of any assumptions or corrections, such as tests of normality and adjustment for multiple comparisons
☐	☒	A full description of the statistical parameters including central tendency (e.g. means) or other basic estimates (e.g. regression coefficient) AND variation (e.g. standard deviation) or associated estimates of uncertainty (e.g. confidence intervals)
☐	☒	For null hypothesis testing, the test statistic (e.g. *F*, *t*, *r*) with confidence intervals, effect sizes, degrees of freedom and *P* value noted<br>*Give P values as exact values whenever suitable.*
☒	☐	For Bayesian analysis, information on the choice of priors and Markov chain Monte Carlo settings
☒	☐	For hierarchical and complex designs, identification of the appropriate level for tests and full reporting of outcomes
☒	☐	Estimates of effect sizes (e.g. Cohen's *d*, Pearson's *r*), indicating how they were calculated

Our web collection on statistics for biologists contains articles on many of the points above.

Software and code

Policy information about availability of computer code

Data collection	BD FACS DIVA software version 9 was used to collect flow cytometry data
Data analysis	Flow Cytometry: Data were collected using Diva version 9 software on BDCanto II and LSR Fortessa. All data were analyzed using Flow Jo software (TreeStar, version 9.7.6 and 9.9.6)<br>Mutiplex-ELISA: Samples were acquired on a BD LSRFortessa™ flow cytometer. Data was analyzed with the LEGENDplex™ Data Analysis Software (LEGENDplex v8.0).<br>Graphs and statistics: GraphPad Prism Version 5 was used to plot graphs, mean with standard error of means was shown on each graph. The statistical significance was performed using two-tailed pairwise or unpaired student's t test.<br>The steps below encompass the standard pre-processing workflow for scRNA-seq data in Seurat package (version 3.1.1) in R (version 3.5.3) {Satija, 2015 #28688}. Genes expressed in less than 3 cells and cells with no more than 50 detected genes were filtered out. After removing unwanted cells from the dataset, we employ a global-scaling normalization method, “LogNormalize”, that normalizes the gene expression measurements for each cell by the total expression, multiplies this by a scale factor (10,000 by default), and log-transforms the result. 3. Then we applied the ScaleData function to mitigate the technical noise and (or) biological sources of variation, which can improve downstream dimensionality reduction and clustering. Highly variable genes screened with FindVariableFeatures function were focused for downstream analysis. Next we perform PCA on the scaled data with RunPCA function. We identify ‘significant’ PCs as those who have a strong enrichment of low p-value genes based on Jackstraw algorithm. As for cell clustering, we first calculate k-nearest neighbors and construct the SNN graph (FindNeighbors), then top 20 PCs were selected for runing FindClusters. tSNE was used as a powerful tool to visualize and explore these datasets. cells within the graph-based clusters determined above should co-localize on the tSNE plot. FindAllMarkers were applied to find markers that define clusters via differential expression. VlnPlot was applied to show expression probability distributions across clusters and FeaturePlot was applied to visualize gene expression on a tSNE plot.

For manuscripts utilizing custom algorithms or software that are central to the research but not yet described in published literature, software must be made available to editors and reviewers. We strongly encourage code deposition in a community repository (e.g. GitHub). See the Nature Research guidelines for submitting code & software for further information.

Data

Policy information about availability of data

All manuscripts must include a data availability statement. This statement should provide the following information, where applicable:

- Accession codes, unique identifiers, or web links for publicly available datasets
- A list of figures that have associated raw data
- A description of any restrictions on data availability

All relevant data are available from the corresponding author. The source data underlying Figures 1f, 1i, 1k, 2a, 2b, 2d, 2h – 2j, 2m – 2o, 3a – 3c, 3e – 3h, 4b, 4d – 4f, 4h – 4j, 4l, 5e, 5g, 5h, 5k, 5m, 6b, 6d, 6e, 6g – 6i, 7a – 7c, 7f, 7h – 7k, 8b, 8c, 8f, 8h, 8j – 8l, 9b – 9d, 9f, 9g, 9i and Supplementary Figures 1d – 1f, 4a, 5a, 5b, 6b, 9b are provided as a Source Data file.

Field-specific reporting

Please select the one below that is the best fit for your research. If you are not sure, read the appropriate sections before making your selection.

- [x] Life sciences
- [ ] Behavioural & social sciences
- [ ] Ecological, evolutionary & environmental sciences

For a reference copy of the document with all sections, see nature.com/documents/nr-reporting-summary-flat.pdf

Life sciences study design

All studies must disclose on these points even when the disclosure is negative.

Sample size	For all experiments except Figure 7d, we have compared at least three testing and control samples and we show either representative data from one experiments or data pooled data from all experiments. The exact numbers are indicated in the figure legends. Figure 7d is representative of two experiments and each experiment has one mixed bone marrow chimeric mouse with bone marrows from both testing and control mice.
Data exclusions	No data was excluded from the analysis
Replication	Experiments were repeated at least three times for most experiments except that Figure 7d and 8l are representative of two experiments, Figure 8f, 8h, 9a - 9d, and 9i are representative of two experiments with triplicates in each experiment. All attempts at replication were successful.
Randomization	Randomization was not applicable in this study. Sex and age matched animals (in many cases littermates) were used for experimental and control groups
Blinding	Most experiments contain test mice and control mice. Researches know the genotypes of the mice before experiment. Blinding was not applicable.

Reporting for specific materials, systems and methods

We require information from authors about some types of materials, experimental systems and methods used in many studies. Here, indicate whether each material, system or method listed is relevant to your study. If you are not sure if a list item applies to your research, read the appropriate section before selecting a response.

Materials & experimental systems

n/a	Involved in the study	
[ ]	[x]	Antibodies
[x]	[ ]	Eukaryotic cell lines
[x]	[ ]	Palaeontology and archaeology
[ ]	[x]	Animals and other organisms
[x]	[ ]	Human research participants
[x]	[ ]	Clinical data
[x]	[ ]	Dual use research of concern

Methods

n/a	Involved in the study	
[x]	[ ]	ChIP-seq
[ ]	[x]	Flow cytometry
[x]	[ ]	MRI-based neuroimaging

Antibodies

Antibodies used	For analysis of mucosal associated invariant T cell compartments and Mucosal associated invariant T cell features were stained with anti-mouse antibodies against the following (Clone name, conjugated fluorescence, manufacturer): CD45.2 (104,APC-Cy7 ,PE-Cy7,PE,BioLegend), CD45.1 (A20, FITC, APC-Cy7, BUV421, BV605, BioLegend), TCRb (H57-597, PE-Cy5, BV510, APC, PE, FITC, BioLegend), CD44 (IM7, BUV737, BD Bioscience, BV510, BioLegend), CD4 (GK1.5, BUV496, BD Bioscience, APC-Cy7, PE-Cy7, BioLegend), CD8a (53-6.7,BV711,BV650,APC-Cy7,PE-Cy7,BioLegend), IFN-g (XMG1.2,PECy7,

PE,BioLegend), IL17A (TC11-18H10.1,PE,PerCP-Cy5.5,BioLegend), T-bet (4B10, BV605, PE-Cy7, BioLegend), RORgt (Q31-378,BV786,BD Bioscience), pAKT S473 (SDRNR,APC, eBioscience or Thermo Fisher Scientific), pS6 (cupk43k,PE-Cy7, eBioscience or Thermo Fisher Scientific), Live/Dead Fixable Violet Dead Cell Stain (BV421, Invitrogen), Fixable Viability Dye eFluor 780 (eBioscience),PLZF (Mags.21F7, Alexa Fluor 488, PE, Invitrogen), ICOS (C398.4A,PE-Cy7,APC-Cy7,BioLegend), CD45(30-F11,BV500,BD Bioscience), CD24 (M1/69, BV605,BUV496, BD Bioscience), CD122 (TM-Beta1, BV711,BD Bioscience), 5-OP-RU-loaded MR1-Tet(APC, PE, kindly provided by the NIH tetramer facility), B220 (RA3-6B2,BV421, FITC, BioLegend), CD11b (M1/70, BV421, FITC,BioLegend), CD11c (N4/8,BV421,FITC,BioLegend), F4/80 (BM8, BV421, FITC BioLegend), TCRgd (GL3,PE,APC,BioLegend), CD19 (6D5, BV421,BioLegend), Ter119 (TER-119,BV421,FITC,BioLegend), Gr1 (RB6-8C5, BV421,FITC, BioLegend), IL-1R1 (clone JAMA-147, Bioton, BioLegend), Streptavidin (FITC, Biolegend), PBS-57-loaded CD1d-Tet (PE,APC,kindly provided by the NIH tetramer facility), Ki-67 (catalog number: 550609, BD Bioscience), PE Isotype Control (MOPC-21, BD Bioscience), pAkt T308 (244F9, Cell Signal), Raptor (24C12, Cell Signal), Rictor (53A2, Cell Signal), Alexa Fluor 568 goat anti-mouse IgG (catalog number: A11031, ThermoFisher), Texas Red goat anti-rabbit IgG (catalog number: T-2767, Molecular Probes), IRF4 (3E4, FITC, Invitrogen), cMAF (symOF1, PE, Invitrogen) and BATF (MBM7C7, PE, Invitrogen).

Validation: We carefully selected antibodies from trustful commercial sources. Antibody validation is based on manufacturers' websites. Details can be found in manufacturers' websites based on information in supplemental table 2, Additionally, we validated antibodies experimentally based on known cell populations and expression levels. we also optimized antibody dilutions experimentally before study.

Animals and other organisms

Policy information about studies involving animals; ARRIVE guidelines recommended for reporting animal research

Laboratory animals: C57BL/6J mice (Stock No. 000664), hCD2-icre mice (Stock No. 027406), Icostm1Mak(Stock No.004859), Tbx21F/F (Stock No.022741), Il2rbfl (Stock No.029657), mTORF/F (Stock No.011009), RaptorF/F (Stock No.029657), RictorF/F (Stock No.020649), CD4Cre (Stock No.017336), CD45.1 (stock No.008451) were obtained from Jackson Laboratory.
CD4Cre mice (stock N. 4196) from Taconic Inc.
TCRJa18-/- mice were kindly provided by Drs. Masaru Taniguchi, Kim Nichols, and Luc Van Kaer and is referenced.
DGKzWT and DGKzdNLS mice were recently reported and are referenced.

Wild animals: N/A

Field-collected samples: N/A

Ethics oversight: Experiments described are approved by the Institutional Animal Care and Use Committee of Duke University

Note that full information on the approval of the study protocol must also be provided in the manuscript.

Flow Cytometry

Plots

Confirm that:

- [x] The axis labels state the marker and fluorochrome used (e.g. CD4-FITC).
- [x] The axis scales are clearly visible. Include numbers along axes only for bottom left plot of group (a 'group' is an analysis of identical markers).
- [x] All plots are contour plots with outliers or pseudocolor plots.
- [x] A numerical value for number of cells or percentage (with statistics) is provided.

Methodology

Sample preparation: We used standard protocols to prepare single-cell suspensions from the thymus,spleen, pLNs, mLNs, Lung and liver of mice

Instrument: Flow cytometry analysis was done using BD FACSCanto II and Fortessa.

Software: FACS data were collected using BD FACS Diva software version 9 and analyzed using Flowjo software version 9.7.6 and 9.9.6. Prism 5/GraphPad software was used for statistical analysis.

Cell population abundance: Large events (about 0 - 2 x10E6 cells) were usually collected to assess MAIT cell populations. Stringent gateing and dumping strategies were used to identify MAIT cells. MAIT cell populations were confirmed with MAIT cell deficient TCRJa18 deficient mice. For sorting MAIT cells, post-sorting purity was 98% determined by FACS.

Gating strategy: Using FSC and SSC to gate on lymphocytes, FSC-A and FSC-H to exclude doublets, use live/dead staining to lineage dump channels to gate on live and Lin- cells. Then use TCRb and Mr1-Tetramer to identify MAIT cells. Gating strategy is shown in supplemental figure 10.

- [x] Tick this box to confirm that a figure exemplifying the gating strategy is provided in the Supplementary Information.
